# Sex Differences in Becoming a Current Electronic Cigarette User, Current Smoker and Current Dual User of Both Products: A Longitudinal Study among Mexican Adolescents

**DOI:** 10.3390/ijerph17010196

**Published:** 2019-12-27

**Authors:** Rosibel Rodríguez-Bolaños, Edna Arillo-Santillán, Inti Barrientos-Gutiérrez, Luis Zavala-Arciniega, Charity A. Ntansah, James F. Thrasher

**Affiliations:** 1 Population Health Research Center, Mexican National Institute of Public Health, Av. Universidad 655 Col. Santa María Ahuacatitlán, Cuernavaca, Morelos 62100, Mexico; edna@insp.mx (E.A.-S.); thrasher@mailbox.sc.edu (J.F.T.); 2 School of Demography, Australian National University, Canberra 2601, Australia; 3 Center for Evaluation and Survey Research, Mexican National Institute of Public Health, Av. Universidad 655 Col. Santa María Ahuacatitlán, Cuernavaca, Morelos 62100, Mexico; inti.barrientos@insp.mx; 4 Department of Epidemiology, University of Michigan, Ann Arbor, MI 48109, USA; luiszavala171409@gmail.com; 5 Department of Health Promotion, Education & Behavior, Arnold School of Public Health, University of South Carolina, Columbia, SC 29208, USA; CNTANSAH@email.sc.edu

**Keywords:** electronic cigarettes, cigarette smoking, Mexico, adolescents, sex difference

## Abstract

This study aimed to assess sex differences in predictors for becoming a current exclusive electronic cigarette (e-cigarette) user, current exclusive smoker, or current dual user (concurrent smoking and e-cigarette use). This longitudinal study included 2399 females and 2177 males who had tried neither cigarettes nor e-cigarettes at baseline and attended 57 middle schools in the three largest cities in Mexico. We estimated multinomial logistic models stratified by sex. At follow-up, the prevalence of current exclusive e-cigarette use was 6.4% for males and 5.5% for females; current exclusive smoking was similar among males (3.6%) and females (3.5%); dual use was 2.4% females and 1.8% males. In the adjusted model, current e-cigarette use among females was associated with baseline current drinking (ARR = 1.85; *p* < 0.05), having a job (ARR = 1.99; *p* < 0.05), higher technophilia (ARR = 1.27; *p* < 0.05), and higher positive smoking expectancies (ARR = 1.39; *p* < 0.05). Among males, only having friends who smoke cigarettes at baseline was a significant predictor of current exclusive e-cigarette use at follow-up (ARR = 1.44; *p* < 0.05). For both sexes, current exclusive smoking at follow-up was associated with baseline current drinking (male ARR = 2.56; *p* < 0.05; female ARR = 2.31; *p* < 0.05) and, among males, only with having a parent who smoked (ARR = 1.64; *p* < 0.05). For both sexes, dual use at follow-up was associated with baseline current drinking (male ARR = 3.52; *p* < 0.005; female ARR = 2.77; *p* < 0.05); among females, with having paid work (ARR = 2.50; *p* < 0.001); and among males, with parental smoking (ARR = 3.20; *p* < 0.05). Results suggested both common and different risk factors by sex, suggesting that interventions may need to consider targeting sex differences.

## 1. Introduction

Electronic cigarette (e-cigarette) use among young people has grown substantially [1,2], even in countries where the importation, distribution, marketing and sales of e-cigarettes are banned, such as Mexico [3,4,5]. E-cigarettes can attract relatively low risk youth to start using nicotine products [6,7], and lead to smoking more harmful combustible cigarettes [8,9,10,11]. Males are more likely to report e-cigarette use than females [11,12,13,14,15,16,17], yet little is known about sex differences in the risk factors for e-cigarette use [18]. Our longitudinal study aimed to assess sex differences in the predictors for current exclusive e-cigarette use, exclusive smoking and dual use (concurrent smoking and e-cigarette use) among middle school adolescents in Mexico who had not tried any tobacco products at baseline.

The prevalence of e-cigarette use among male adolescents is consistently higher than among females [18]. This includes Global Youth Tobacco Survey data from Latin American countries, except in Chile (9.9% males and 13.7% female), where both smoking and e-cigarettes is higher among females [19]. Even in countries that ban e-cigarettes, use appears to be relatively high, as in Mexico where 12% of middle schoolers were current users in 2016 [5]. Furthermore, where combined rates of e-cigarette or cigarette use among adolescents has been evaluated (in the US), the prevalence is also higher among males than females [20]. In fact, the prevalence of any tobacco product use is generally higher among males than females [21], although e-cigarettes marketing strategies that target females and youth may ultimately change this pattern [22,23].

Most risk factors for e-cigarette use are similar to those for smoking [6,7,11]. Nevertheless, some risk factors appear specific to e-cigarette use. For example, one unique risk factor is “technophilia,” or the appeal of using new technologies, which appears higher among females than males, likely due to their greater social media use [5,24]. Internet and social media use may not only reflect technophilia, but also exposure to social media ads that promote e-cigarettes among youth [7]. Indeed, time spent online and using social media appear associated with awareness of and searching for information about e-cigarettes [25,26]. Other studies indicate that friends and family use of tobacco products has a stronger influence on e-cigarette susceptibility [24], initiation [27] and use [28] among females than males. While very few studies have explicitly evaluated sex differences in e-cigarette risk factors, some evidence suggests that female e-cigarette use is driven more by social network, social media, and internet influences than for males.

The current longitudinal study examined the baseline risk factors for becoming a current user of e-cigarettes, cigarettes, and dual use by sex. Research cited above suggests that e-cigarette risk factors involving technophilia, internet use, and tobacco product use among parents and friends may be stronger among females than males. It is not clear how such risk factors will influence e-cigarettes use in countries where they are illegal and therefore potentially more stigmatized, as in Mexico and, increasingly, around the world [29]. Nevertheless, consumption and purchase of e-cigarettes in Mexico and many other countries are not illegal, which may mitigate cross-country differences in risk factors. Understanding whether the predictors of e-cigarette use differ by sex can inform intervention strategies that consider sex when aiming to address the epidemic of e-cigarette use.

## 2. Materials and Methods

### 2.1. Study Population

We used data from a longitudinal study of students from 57 randomly selected public middle schools in the three largest cities in Mexico (Mexico City, Guadalajara, and Monterrey). A detailed description is available of the multi-stage school selection procedure, where the overall baseline response rate for all eligible first-year students in February and March 2015 was 84.0% [7]. The follow-up survey in October and November 2016 was administered in the last year of middle school, where students could participate whether they were surveyed at baseline or not. Passive parental consent was used, with students providing active assent. Study protocols were approved by the Ethics Committee of Mexican National Institute of Public Health (acronym in Spanish INSP; CI 1104; copies of the survey can be solicited from the corresponding author).

The sample included 6536 students who were surveyed at baseline and follow-up. After excluding students who tried combustion cigarettes or e-cigarettes at baseline (n = 1685) and those with missing covariates (n = 275), the final analytic sample consisted of 2399 females and 2177 males.

### 2.2. Measure

#### 2.2.1. Baseline Covariates

Socio-demographic characteristics included sex, age (i.e., 11 to 12 or 13 to 15 years old), and highest educational attainment of either parent (i.e., primary, middle, high school or more, unknown). Personal risk factors included a 4-item sensation seeking scale (e.g., “I like to do frightening things”; α = 0.80), previously validated for Mexican youth [30]; a 4-item measure of positive smoking expectancies (e.g., “Smoking makes me feel good”; α = 0.89) [31]; alcohol use (i.e., never used; tried but not in the last 30 days; current drinking in the last 30 days) [32]; ever use of drugs, focused on the most commonly used illegal drugs in Mexico (i.e., marijuana, cocaine) [7,33]; technophilia measured with three-items about technology ownership (i.e., computers, smartphones and videogames) [7]; frequency of digital social media (e.g., Facebook, Snapchat, Twitter, Instagram) use in the past month (no use; occasional use; frequent use); parental rules about internet use (yes/no); current working status (i.e., “Do you have any work for which you are paid?”); and maternal monitoring/control (e.g., “She tells me what time I have to be home”; α = 0.70) using three averaged items for each dimension [34]. Social network smoking behavior included: parental smoking (either parent vs. neither); smoking among at least one of their five best friends (none vs. one or more).

#### 2.2.2. Dependent Variables at Follow-Up

At follow-up, current cigarette use was determined by asking students: “During the past 30 days, on how many days did you smoke cigarettes?”, with those who reported smoking at least once defined as current smokers. Similarly, for current e-cigarette use, students were asked: “During the past 30 days, on how many days did you use e-cigarettes?” Those who reported using e-cigarettes at least once in the prior 30 days were defined as current users. These two categories were used to divide the sample into: (1) Non-current user of either product; (2) Exclusive e-cigarettes users; (3) Exclusive smokers; and (4) Dual users, who concurrently smokers and e-cigarettes users.

### 2.3. Analysis

Within each tobacco product use group at follow-up (i.e., non-current users; exclusive e-cigarette users; exclusive smokers; dual users), descriptive statistics were evaluated for all study variables. We estimated multinomial logistic models regressing tobacco product use (non-current users of either cigarettes or e-cigarettes = reference group; exclusive e-cigarette users; exclusive cigarette users; and dual users) stratified by sex. Both bivariate and adjusted models were estimated for the covariates described above. As a sensitivity analysis, we excluded the adolescents that responded that they used in cigarettes or e-cigarettes in the last 30 days, but not in their lifetime. Furthermore, we used the VCE cluster command that allows getting robust standard errors that adjusted for the correlations among students at the school level (57 middle schools). The analyses were performed using STATA 15 (StataCorp. 2017. Stata Statistical Software: Release 15. College Station, TX, USA). In the specification of the multinomial models, STATA provides an option to estimate Relative Risk Ratios (RRRs), which we consider was adequate since our analyses were longitudinal

## 3. Results

About half of the sample was female (52.4%). At baseline, most students were 11 to 12 years old (males 65.0% and female 67.2%), and more males (55.0%) than females (45.0%) had a job. Characteristics of the sample by sex are presented in the Table 1.

### 3.1. Current Exclusive e-Cigarette Use at Follow-Up

At follow-up, the prevalence of exclusive current e-cigarette use was 6.4% among males and 5.5% among females. In adjusted multinomial models for females, where exclusive e-cigarettes were compared to noncurrent users of either product, baseline having a job (Adjusted Relative Risk Ratios or ARR: 1.99, 95% CI 1.18, 3.37), current drinking (ARR: 1.85, 95% CI 1.09, 3.15), higher technophilia (ARR: 1.27, 95% CI 1.05, 1.55), and higher positive smoking expectancies (ARR: 1.39, 95% CI 1.10, 1.74) were associated with becoming an exclusive e-cigarettes user (Table 2). Among males, only having friends who smoke was an independent, statistically significant predictor of exclusive e-cigarette use (ARR: 1.44, 95% CI 1.02, 2.01; see Table 3).

### 3.2. Current Exclusive Smoking at Follow-Up

At follow-up, exclusive current smoking was similar among males (3.6%) and females (3.5%). In adjusted multinomial models for both sexes, predictors of smoking included current drinking (male ARR: 2.56, 95% CI 1.50, 4.38; female ARR: 2.31, 95% CI 1.24, 4.31; see Table 2 and Table 3). Having a parent who smoked cigarettes (ARR: 1.64, 95% CI 1.07, 2.49) predicted smoking among males only (Table 3).

### 3.3. Dual Use of e-Cigarettes and Smoking at Follow-Up

The prevalence of dual use at follow-up was 2.4% among females and 1.8% among males. In adjusted models for both sexes, baseline current drinking predicted dual use (male ARR: 3.52, 95% CI 1.66, 7.68; female ARR: 2.77, 95% CI 1.11, 6.57; see Table 2 and Table 3). For females, having a job predicted smoking (ARR: 2.50, 95% CI 1.62, 0.86) (Table 2), and, among males, parental smoking predicted smoking (ARR: 3.20, 95% CI 1.40, 7.34; see Table 3). The findings from the sensitivity analyses were in the same direction and significance as the resulted reported here (see Appendix A).

## 4. Discussion

In general, we found negligible differences between males and females in the incidence of becoming a user of e-cigarettes, cigarettes or both, although risk factors differed somewhat by sex. Consistent with prior research [18], including in Latin America [19], we found that becoming a current exclusive e-cigarette user was somewhat higher among males (6.4%) than females (5.5%); however, this relatively small difference was balanced out by the slightly lower prevalence of becoming a current dual user amongst males (1.8%) than females (2.4%). Overall, the differences between males and females were negligible, suggesting that the likelihood of using tobacco products is becoming similar across sexes in Mexico.

The number of differences in the risk factors for tobacco product use are notable. For males, friend and parental smoking was a predictor of current smoking and e-cigarette use, which is consistent with results from study that did not stratify analyses by sex [28,35]. However, this result contrasts with prior, cross-sectional studies Mexico [24] and longitudinal in Finland [27], in which tobacco product use among family were associated with greater susceptibility to and initiation of e-cigarette use with female. The reasons for these differences in risk factors are not clear; however, it may be because our baseline measures of tobacco product use amongst network members was limited to smoking, not e-cigarette use. This was because we had assumed that e-cigarette use would be uncommon given the ban in Mexico and the young age of the sample (12.5 years at baseline). In our subsequent, cross-sectional research on the follow-up survey from this sample, we found evidence for product specific network influences on e-cigarette use susceptibility amongst never users, including stronger effects of peer and parent e-cigarette use on females than males [24]. Future longitudinal research should evaluate whether network use of specific product types influences specific product use amongst youth, including by sex.

For both males and females, baseline alcohol use predicted current smoking and dual use, as in other studies [14,36], but we found that it predicted e-cigarette use only among females. Risk for polysubstance use among female is increasing [37,38], and little is known about this topic. These data suggest that efforts to prevent e-cigarette use should target a variety of substances, perhaps by focusing on common risk factors, such as social influence and building refusal skills [39]. In so doing, interventions may need to consider how gender roles influence substance use.

We found that females who reported having a job were more likely to become current e-cigarettes users and dual users, whereas this association was not found among males. In prior studies, this association has been explained by focusing on how greater economic resources allow for purchasing e-cigarettes, which can be relatively expensive compared to cigarettes [16,35,40]; however, none of these studies evaluated whether this association differs by sex. Adolescents who report having a personal income show independence behavior, gain relatively more autonomy from parents and possibly have less parental oversight. Indeed, a study conducted in different European cities found a strong relationship between personal income and smoking [41]. Furthermore, workplaces often provide opportunities for adolescents to socialize with adults, who may be more likely to use tobacco products. The idea of independence, equality, and autonomy that the tobacco industry has used to promote its products, including in its female-targeted promotions, may be more effective among young females than males.

Our proxy measure of technophilia predicted current e-cigarette use, whether exclusive or dual use, but only among females. It is not clear why technophilia would be a risk factor specifically for female. As discussed in prior research on this topic [5,7], this may be due to greater social media use and, through this, more exposure to online advertising; however, we found that frequency of social media use was unassociated with any outcome for either sex. Hence, technophilia appears to be an independent risk factor for e-cigarette use among adolescents in Mexico [5,7] and should be explored in studies conducted in other countries, including those with contrasting e-cigarette policies.

Our study has some potential limitations. We collected data from students in only three cities, but these cities are the largest in Mexico and therefore provide a reasonable approximation of other urban populations, where about three-quarters of all Mexicans live [10]. However, results may not generalize to rural areas of Mexico, where access to e-cigarettes may be quite different. Furthermore, the baseline survey was developed to evaluate media influences on smoking initiation; hence, we included only a few questions that were specific to e-cigarette awareness and behavior at baseline because of our incorrect assumption that e-cigarette use would be low given the young age of the sample, their relatively recent introduction, and their illegality. As a result, we did not fully evaluate potential media, network, or psychosocial influences on uptake, and our results may therefore suffer from omitted variable bias. We also may have missed important marketing exposures and social influences that emerged in the interim between survey waves, which covers a time when the market for e-cigarettes was rapidly evolving. Finally, the study was not designed to evaluate substance use from a gender perspective, which would have involved examining potential differences related to social facilitation, appearance, weight control and management of stress and mood [42]. Future research should more fully consider gender differences in the mechanisms that may explain e-cigarette use.

## 5. Conclusions

This study—conducted in the context of a country that bans e-cigarette sales and marketing, but not their consumption—found that e-cigarette use was surprisingly high among both male and female middle school students, having eclipsed the incidence of smoking in both groups. We also found more differences than similarities in the risk factors for current e-cigarette use across males and females. In particular, parent and friend smoking was a risk factor among males but not females, whereas working, technophilia and alcohol use were risk factors for females but not males. For both, drinking was a strong risk factor for current smoker and current dual user. Future research and actions that reduce health disparities using a gender approach should be considered. Furthermore, it will be critical to monitor potential health effects from e-cigarette use, such as EVALI (e-cigarette, or vaping, product use-associated lung injury), which appears due to consumption of black market cannabis oils using vaping devices. In Mexico at least one fatal case has been documented, though this number may grow due to the unregulated, illegal nature of the e-cigarette market.

## Figures and Tables

**Table 1:** Prevalence of characteristic of sample: Non-current users, current e-cigarette users, current smokers and current dual users by sex, Mexico 2015–2016.

Female	Non-Current User of Nicotine Products	Current e-Cigarette Users	Current Smokers	Current Dual User (i.e., Smoke and Use e-Cigarettes)
Total (n = 2399)	(n = 2126)	(n = 132)	(n = 84)	(n = 57)
Age years				
11 to 12	67.7%	60.6%	66.7%	64.9%
13 to 15	32.3%	39.4%	33.3%	35.1%
Job				
No	89.1%	80.3%	88.1%	73.7%
Yes	10.9%	19.8%	11.9%	26.3%
Parental education				
Primary	16.0%	15.9%	17.9%	21.0%
Middle School	39.7%	40.9%	44.1%	36.8%
High School or more	37.0%	36.4%	34.5%	36.8%
Unknown	7.4%	6.8%	3.6%	5.3%
Use alcohol				
Never	67.0%	54.6%	38.1%	40.4%
Tried	24.2%	27.3%	44.1%	29.8%
Current drinking	8.9%	18.2%	17.9%	29.8%
Ever use of drug				
No	97.8%	97.0%	95.2%	89.5%
Yes	2.2%	3.0%	4.8%	10.5%
Sensation seeking, mean (sd)	2.56 (0.99)	2.81 (1.15)	3.04 (0.91)	3.18 (1.12)
Technophilia index, mean (sd)	1.77 (0.99)	2.02 (0.98)	1.81 (1.00)	1.87 (1.01)
Digital social media use				
No use	14.8%	12.9%	13.1%	17.5%
Ocassional use	22.1%	18.2%	19.1%	15.8%
Frecuent use	63.1%	68.9%	67.9%	66.7%
Parental rules about internet use				
Yes	76.6%	71.2%	66.7%	59.6%
No	23.4%	28.8%	33.3%	40.4%
Positive smoking expectancies, mean (sd)	1.49 (0.69)	1.76 (0.96)	1.80 (0.85)	1.96 (1.04)
Friends smoking				
None	76.9%	67.4%	66.7%	59.7%
One or more	23.1%	32.6%	33.3%	40.4%
Parental smoking				
Neither	62.9%	61.4%	47.6%	56.1%
Either parent	37.1%	38.6%	52.4%	43.8%
Maternal monitoring/control, mean (sd)	4.08 (0.91)	4.04 (0.95)	3.8 (1.03)	3.8 (1.06)
**Male**	**Non-Current User of Nicotine Products**	**Current e-Cigarette Users**	**Current Smokers**	**Current Dual User** **(i.e., Smoke and Use e-Cigarettes)**
Total (n = 2177)	(n = 1920)	(n = 139)	(n = 79)	(n = 39)
Age years				
11 to 12	64.8%	69.1%	62.0%	64.1%
13 to 15	35.2%	39.9%	38.0%	35.9%
Job				
No	84.5%	82.0%	81.0%	79.5%
Yes	15.4%	18.0%	19.0%	20.5%
Parental education				
Primary	17.8%	20.1%	22.8%	23.1%
Middle School	34.8%	42.5%	39.2%	30.8%
High School or more	37.3%	31.7%	30.4%	43.6%
Unknown	10.0%	5.8%	7.6%	2.6%
Use alcohol				
Never	62.5%	52.5%	41.8%	35.9%
Tried	27.8%	30.9%	35.4%	35.9%
Current drinking	47.3%	37.5%	38.0%	46.2%
Ever use of drug				
No	96.7%	94.2%	91.1%	89.7%
Yes	3.3%	5.8%	8.9%	10.2%
Sensation seeking, mean (sd)	2.74 (1.05)	2.95 (1.06)	2.93 (1.04)	3.03 (1.07)
Technophilia index, mean (sd)	1.73 (1.60)	1.88 (1.01)	1.80 (0.97)	1.76 (1.13)
Digital social media use				
No use	20.4%	18.7%	21.5%	15.4%
Ocassional use	27.8%	28.1%	19.0%	10.3%
Frecuent use	51.8%	53.2%	59.5%	74.4%
Parental rules about internet use				
Yes	69.3%	64.0%	65.9%	51.3%
No	30.7%	36.0%	34.2%	48.7%
Positive smoking expectancies, mean (sd)	1.60 (0.78)	1.77 (0.90)	1.78 (0.86)	1.80 (0.85)
Friends smoking				
None	78.7%	68.4%	63.3%	71.8%
One or more	21.3%	31.7%	36.7%	28.2%
Parental smoking				
Neither	66.5%	55.4%	50.6%	35.9%
Either parent	33.5%	44.6%	49.4%	64.1%
Maternal monitoring/control, mean (sd)	4.10 (0.92)	4.07 (0.99)	4.00 (0.97)	4.05 (1.22)

**Table 2:** Risk factors for exclusive e-cigarette use, exclusive smoking, and dual use among females, 2015–2016.

Female (n = 2399)	Current e-Cigarette Users			Current Smokers			Current Dual User (i.e., Smoke and Use e-Cigarettes)		
		ARR	(95% CI)		ARR	(95% CI)		ARR	(95% CI)
Age years									
11 to 12	5.0%	1.00		3.5%	1.00		2.3%	1.00	
13 to 15	6.6%	1.31	(0.94–1.83)	3.6%	0.92	(0.54–1.57)	2.5%	0.98	(0.54–1.78)
Job									
No	5.0%	1.00		3.5%	1.00		2.0%		
Yes	9.2%	1.99 *	(1.18–3.37)	3.5%	0.99	(0.52–1.89)	5.3%	2.50 ***	(1.62–3.86)
Parental education									
Primary	5.4%	1.00		3.9%	1.00		3.1%	1.00	
Middle School	5.7%	1.19	(0.75–1.90)	3.9%	1.14	(0.60–2.16)	2.2%	1.07	(0.55–2.07)
High School or more	5.4%	1.12	(0.68–1.83)	3.3%	0.96	(0.52–1.76)	2.4%	1.13	(0.52–2.46)
Unknown	5.3%	1.04	(0.49–2.20)	1.7%	0.55	(0.17–1.73)	1.7%	0.82	(0.17–3.87)
Use alcohol									
Never	4.6%	1.00		2.1%	1.00		1.5%	1.00	
Tried	6.0%	1.11	(0.70–1.76)	6.1%	2.31 **	(0.31–4.08)	2.8%	1.34	(0.64–2.78)
Current drinking	9.9%	1.85 *	(1.09–3.15)	6.2%	2.31 **	(1.24–4.31)	7.0%	2.77 *	(1.17–6.57)
Ever use of drug									
No	5.5%	1.00		3.4%	1.00		2.2%	1.00	
Yes	6.6%	0.82	(0.27–2.43)	6.6%	1.23	(0.39–3.87)	9.8%	2.15	(0.83–5.59)
Sensation seeking, mean (sd)		1.03	(0.85–1.25)		1.27	(0.97–1.67)		1.35	(0.94–1.93)
Technophilia index, mean (sd)		1.27 *	(1.05–1.55)		0.96	(0.74–1.24)		1.04	(0.79–1.38)
Digital social media use									
No use	4.8%	1.00		3.1%	1.00		2.8%	1.00	
Ocassional use	4.6%	0.93	(0.49–1.76)	3.1%	1.03	(0.49–2.18)	1.7%	0.65	(0.32–1.30)
Frecuent use	6.0%	1.08	(0.58–1.99)	3.7%	1.12	(0.56–2.23)	2.5%	0.95	(0.41–2.17)
Parental rules about internet use									
Yes	5.2%	1.00		3.1%	1.00		1.9%	1.00	
No	6.5%	1.18	(0.79–1.76)	4.8%	1.29	(0.80–2.08)	3.9%	1.54	(0.85–2.78)
Positive smoking expectancies, mean (sd)		1.39 *	(1.10–1.74)		1.23	(0.92–1.64)		1.31	(0.93–1.85)
Friends smoking									
None	4.9%	1.00		3.1%	1.00		1.9%	1.00	
One or more	7.4%	1.21	(0.84–1.73)	4.8%	1.11	(0.67–1.86)	3.9%	1.39	(0.72–2.67)
Parental smoking									
Neither	5.4%			2.7%			2.2%		
Either parent	5.6%	0.94	(0.67–1.31)	4.8%	1.55	(0.98–2.44)	2.8%	1.08	(0.59–1.98)
Maternal monitoring/control, mean (sd)		1.03	(0.82–1.28)		0.81	(0.65–1.02)		0.84	(0.65–1.09)

*p* value < 0.05 *, < 0.005 **, < 0.001 ***. Estimation involved multinomial models where the reference group was non-current use of e-cigarettes or cigarettes.

**Table 3:** Risk factors for exclusive e-cigarette use, exclusive smoking, and dual use among males, 2015–2016.

Male (n = 2.177)	Current e-Cigarette Users			Current Smokers			Current Dual User (i.e., Smoke and Use e-Cigarettes)		
		ARR	(95% CI)		ARR	(95% CI)		ARR	(95% CI)
Age									
11 to 12	6.8%	1.00		3.5%	1.00		1.8%	1.00	
13 to 15	5.6%	0.80	(0.53–1.19)	3.9%	1.03	(0.69–1.54)	1.8%	0.89	(0.48–1.67)
Job									
No	6.2%	1.00		3.5%	1.00		1.7%	1.00	
Yes	7.2%	1.05	(0.60–1.84)	4.3%	1.08	(0.61–1.91)	2.3%	1.22	(0.48–3.07)
Parental education									
Primary	7.1%	1.00		4.5%	1.00		2.3%	1.00	
Middle School	7.7%	1.21	(0.72–2.06)	4.0%	1.07	(0.62–1.86)	1.6%	0.83	(0.35–1.98)
High School or more	5.5%	0.88	(0.52–1.49)	3.0%	0.83	(0.43–1.60)	2.1%	1.10	(0.37–3.28)
Unknown	3.9%	0.61	(0.24–1.57)	2.9%	0.82	(0.33–2.05)	0.5%	0.22	(0.02–2.18)
Use alcohol									
Never	5.5%	1.00		2.5%	1.00		1.1%	1.00	
Tried	6.9%	1.09	(0.71–1.69)	4.5%	1.59	(0.85–2.98)	2.3%	1.67	(0.70–3.98)
Current drinking	9.6%	1.48	(0.91–2.39)	7.5%	2.56 *	(1.50–4.38)	4.6%	3.52 **	(1.66–7.48)
Ever use of drug									
No	6.3%	1.00		3.4%	1.00		1.7%	1.00	
Yes	9.8%	1.24	(0.57–2.70)	8.5%	1.77	(0.70–4.49)	4.9%	2.44	(0.90–6.61)
Sensation seeking, mean (sd)		1.07	(0.90–1.28)		0.99	(0.80–1.23)		1.01	(0.69–1.48)
Technophilia, mean (sd)		1.15	(0.93–1.42)		1.02	(0.81–1.28)		0.85	(0.55–1.32)
Digital social media use									
No use	5.9%	1.00		3.9%	1.00		1.4%	1.00	
Ocassional use	6.6%	1.08	(0.62–1.86)	2.5%	0.64	(0.29–1.39)	0.7%	0.54	(0.15–1.97)
Frecuent use	6.5%	1.05	(0.70–1.57)	4.1%	1.12	(0.63–1.97)	2.5%	2.49	(0.92–6.73)
Parental rules about internet use									
Yes	6.0%	1.00		3.5%	1.00		1.3%	1.00	
No	7.3%	1.18	(0.80–1.75)	3.9%	1.02	(0.59–1.76)	2.8%	1.95	(0.97–3.91)
Positive smoking expectancies, mean (sd)		1.10	(0.87–1.40)		1.02	(0.77–1.34)		1.01	(0.63–1.61)
Friends smoking									
None	5.6%	1.00		3.0%	1.00		1.7%	1.00	
One or more	8.9%	1.44 *	(1.02–2.01)	5.9%	1.66	(0.99–2.77)	2.2%	0.96	(0.42–2.19)
Parental smoking									
Neither	5.5%	1.00		2.8%	1.00		1.0%		
Either parent	8.1%	1.42	(0.95–2.12)	5.1%	1.64 *	(1.07–2.49)	3.3%	3.20 *	(1.40–7.34)
Maternal support, mean (sd)		1.01	(0.82–1.24)		0.98	(0.75–1.27)		1.11	(0.67–1.83)

*p* value < 0.05 *, < 0.005 **, < 0.001 ***. Estimation involved multinomial models where the reference group was non-current use of e-cigarettes or cigarettes.

## Appendix A

**Table A1:** Sensitivity analyses among females.

Female	Current e-Cigarette User		Current Smokers		Current Dual User	
Total (n = 2390)	ARR	95% CI	ARR	95% CI	ARR	95% CI
Age years						
13 to 15	1.38	(0.99–1.93)	0.88	(0.52–1.51)	1.06	(0.59–1.90)
Job						
Yes	1.86	(1.05–3.29)	0.99	(0.53–1.86)	2.77	(1.73–4.45)
Parental education						
Middle school	1.25	(0.75–2.07)	1.15	(0.60–2.20)	1.26	(0.64–2.49)
High School or more	1.16	(0.69–1.97)	0.98	(0.54–1.77)	1.39	(0.65–2.99)
Unknwon	1.01	(0.44–2.33)	0.36	(0.08–1.56)	0.98	(0.21–4.66)
Use alcohol						
Tried	1.12	(0.71–1.77)	2.37	(1.33–4.21)	1.40	(0.67–2.95)
Current drinking	2.00	(1.14–3.50)	2.36	(1.26–4.42)	3.01	(1.25–7.27)
Ever use of drug						
Yes	0.66	(0.16–2.65)	1.24	(0.39–3.91)	1.34	(0.40–4.54)
Sensation seeking	1.03	(0.85–1.26)	1.28	(0.97–1.68)	1.39	(0.97–1.99)
Technophilia index	1.29	(1.06–1.57)	0.96	(0.74–1.25)	0.98	(0.75–1.26)
Digital social media use						
Ocassional use	0.98	(0.49–1.93)	1.05	(0.50–2.20)	0.82	(0.39–1.73)
Frecuent use	1.13	(0.59–2.16)	1.10	(0.56–2.14)	1.24	(0.49–3.10)
Parental rules about internet use						
No	1.17	(0.77–1.76)	1.31	(0.81–2.13)	1.56	(0.87–2.79)
Positive smoking expectancies	1.35	(1.07–1.71)	1.22	(0.91–1.63)	1.28	(0.90–1.84)
Friends smoking						
One or more	1.29	(0.90–1.84)	1.12	(0.67–1.87)	1.29	(0.64–2.59)
Parental smoking						
Yes	0.92	(0.66–1.29)	1.58	(1.00–2.50)	1.10	(0.59–2.04)
Maternal monitoring/control	1.06	(0.85–1.32)	0.82	(0.65–1.02)	0.83	(0.64–1.09)

**Table A2:** Sensitivity analyses among males.

Male	Current e-Cigarette User		Current Smokers		Current Dual User	
Total (n = 2162)	ARR	CI 95%	ARR	CI 95%	ARR	CI 95%
Age years						
13 to 15	0.81	(0.55–1.21)	1.06	(0.71–1.59)	0.93	(0.52–1.69)
Job						
Yes	1.06	(0.62–1.83)	1.08	(0.61–1.93)	1.27	(0.53–3.04)
Parental education						
Middle school	1.13	(0.64–2.01)	1.02	(0.58–1.79)	0.99	(0.44–2.20)
High School or more	0.87	(0.49–1.52)	0.82	(0.43–1.56)	1.37	(0.49–3.85)
Unknwon	0.49	(0.18–1.31)	0.80	(0.32–1.97)	0.25	(0.02–2.53)
Use alcohol						
Tried	1.16	(0.75–1.81)	1.62	(0.86–3.06)	1.84	(0.78–4.35)
Current drinking	1.46	(0.86–2.46)	2.50	(1.45–4.31)	3.83	(1.72–8.54)
Ever use of drug						
Yes	1.26	(0.55–2.86)	1.49	(0.60–3.70)	2.51	(0.94–6.70)
Sensation seeking	1.13	(0.94–1.35)	1.00	(0.81–1.24)	1.10	(0.77–1.56)
Technophilia index	1.11	(0.88–1.40)	1.00	(0.81–1.25)	0.87	(0.55–1.37)
Digital social media use						
Ocassional use	1.14	(0.62–2.11)	0.61	(0.27–1.40)	0.53	(0.14–1.97)
Frecuent use	1.15	(0.72–1.82)	1.15	(0.65–2.04)	2.31	(0.84–6.39)
Parental rules about internet use						
No	1.23	(0.82–1.83)	1.05	(0.61–1.82)	1.82	(0.94–3.54)
Positive smoking expectancies	1.09	(0.86–1.39)	0.99	(0.75–1.32)	1.01	(0.62–1.65)
Friends smoking						
One or more	1.24	(0.84–1.82)	1.60	(0.96–2.68)	0.83	(0.38–1.78)
Parental smoking						
Either parent	1.41	(0.94–2.11)	1.70	(1.10–2.62)	3.48	(1.57–7.68)
Maternal monitoring/control	1.04	(0.85–1.28)	0.97	(0.75–1.26)	1.05	(0.64–1.71)

## References

[1] Academy of Sciences Engineering and Medicine (2018). Public Health Consequences of E-Cigarettes.

[2] U.S. Department of Health and Human Services (2016). E-Cigarette Use among Youth and Young Adults: A Report of the Surgeon General.

[3] Ley General para el Control del Tabaco (2008). Artículo 16, Apartado VI. Diario Oficial de la Federación. 30/04/2008.

[4] Zavala-Arciniega L., Rodríguez-Andrade M.A., Reynales-Shigematsu L.M., Lozano P., Arillo-Santillán E., Thrasher J.F. (2018). Patterns of awareness and use of electronic cigarettes in Mexico, a middle-income country that bans them: Results from a 2016 national survey. Prev. Med..

[5] Barrientos-Gutierrez I., Lozano P., Arillo-Santillan E., Morello P., Mejia R., Thrasher J.F. (2019). “Technophilia”: A new risk factor for electronic cigarette use among early adolescents?. Addict. Behav..

[6] Wills T.A., Knight R., Williams R.J., Pagano I., Sargent J.D. (2015). Risk factors for exclusive e-cigarette use and dual e-cigarette use and tobacco use in adolescents. Pediatrics.

[7] Thrasher J.F., Abad-Vivero E.N., Barrientos-Gutiérrez I., Pérez-Hernández R., Reynales-Shigematsu L.M., Mejía R., Sargent J.D. (2016). Prevalence and correlates of e-cigarette perceptions and trial among early adolescents in Mexico. J. Adolesc. Health.

[8] Kwon E., Seo D.C., Lin H.-C., Che Z. (2018). Predictors of youth e-cigarette use susceptibility in a U.S. nationally representative sample. Addict. Behav..

[9] Soneji S., Barrington-Trimis J.L., Wills T.A., Leventhal A., Unger J.B., Gibson L.A., Yang J., Primack B.A., Andrews J.A., Miech R.A. (2017). Association between initial use of e-cigarettes and subsequent cigarette smoking among adolescents and young adults a systematic review and meta-analysis. JAMA Pediatr..

[10] Lozano P., Barrientos-Gutierrez I., Arillo-Santillan E., Morello P., Mejia R., Sargent J.D., Thrasher J.F. (2017). A longitudinal study of electronic cigarette use and onset of conventional cigarette smoking and marijuana use among Mexican adolescents. Drug Alcohol Depend..

[11] Leventhal A.M., Strong D.R., Kirkpatrick M.G., Unger J.B., Sussman S., Riggs N.R., Stone M.D., Khoddam R., Samet J.M., Audrain-McGovern J. (2015). Association of electronic cigarette use with initiation of combustible tobacco product smoking in early adolescence. JAMA.

[12] Hamilton H.A., Ferrence R., Boak A., Schwartz R., Mann R.E., O’Connor S., Adlaf E.M. (2015). Ever Use of Nicotine and Nonnicotine Electronic Cigarettes among High School Students in Ontario, Canada. Nicotine Tob. Res..

[13] Babineau K., Taylor K., Clancy L. (2015). Electronic Cigarette Use among Irish Youth: A Cross Sectional Study of Prevalence and Associated Factors. PLoS ONE.

[14] Hughes K., Bellis M.A., Hardcastle K.A., McHale P., Bennett A., Ireland R., Pike K. (2015). Associations between e-cigarette access and smoking and drinking behaviours in teenagers. BMC Public Health.

[15] Porter L., Duke J., Hennon M., Dekevich D., Crankshaw E., Homsi G., Farrelly M. (2015). Electronic Cigarette and Traditional Cigarette Use among Middle and High School Students in Florida, 2011–2014. PLoS ONE.

[16] White J., Li J., Newcombe R., Walton D. (2015). Tripling use of electronic cigarettes among New Zealand adolescents between 2012 and 2014. J. Adolesc. Health.

[17] Goniewicz M.L., Zielinska-Danch W. (2012). Electronic cigarette use among teenagers and young adults in Poland. Pediatrics.

[18] Kong G., Kuguru K.E., Krishnan-Sarin S. (2017). Gender differences in US adolescent e-cigarette use. Curr. Addict. Rep..

[19] Centers for Disease Control and Prevention National Center for Chronic Disease Prevention and Health Promotion, Office of Smoking and Health, Global Tobacco Surveillance System Data (GTSSData). https://www.cdc.gov/tobacco/global/gtss/gtssdata/index.html.

[20] Barrington-Trimis J.L., Urman R., Leventhal A.M., Gauderman W.J., Boley Cruz T., Gilreath T.D., Howland S., Unger J.B., Berhane K., Samet J.M. (2016). E-cigarettes, cigarettes, and the prevalence of adolescent tobacco use. Pediatrics.

[21] Higgins S.T., Kurti A.N., Redner R., White T.J., Gaalema D.E., Roberts M.E., Doogan N.J., Tidey J.W., Miller M.E., Stanton C.A. (2015). A Literature Review on Prevalence of Gender Differences and Intersections with Other Vulnerabilities to Tobacco Use in the United States, 2004–2014. Prev. Med..

[22] Basch C.H., Mongiovi J., Hillyer G.C., Ethan D., Hammond R. (2016). An Analysis of Electronic Cigarette and Cigarette Advertising in US Women’s Magazines. Int. J. Prev. Med..

[23] Drope J., Schluger N., Cahn Z., Drope J., Hamill S., Islami F., Liber A., Nargis N., Stoklosa M. (2018). The Tobacco Atlas.

[24] Lorenzo-Blanco E.I., Unger J.B., Thrasher J.F. E-cigarette Use Susceptibility among Youth in Mexico: The Roles of Remote Acculturation, Parenting Behaviors, Media Use, and Gender. J. Adolesc..

[25] Emery S.L., Vera L., Huang J., Szczypka G. (2014). Wanna know about vaping? Patterns of message exposure, seeking and sharing information about e-cigarettes across media platforms. Tob. Control.

[26] Grana R.A., Ling P.M. (2014). “Smoking revolution”: A content analysis of electronic cigarette retail websites. Am. J. Prev. Med..

[27] Kinnunen J.M., Ollila H., Minkkinen J., Lindfors P.L., Rimpelä A.H. (2018). A Longitudinal Study of Predictors for Adolescent Electronic Cigarette Experimentation and Comparison with Conventional Smoking. Int. J. Environ. Res. Public Health.

[28] Hanewinkel R., Isensee B. (2015). Risk factors for e-cigarette, conventional cigarette, and dual use in German adolescents: A cohort study. Prev. Med..

[29] Institute for Global Tobacco Control (2019). Country Comparison Database—Electronic Cigarette Regulations. Institute for Global Tobacco Control.

[30] Thrasher J.F., Sargent J.D., Huang L., Arillo-Santillán E., Dorantes-Alonso A., Pérez-Hernández R. (2009). Does exposure to smoking in films promote smoking in middle-income countries? A longitudinal study among Mexican adolescents. Cancer Epidemiol. Biomark. Prev..

[31] Wahl S.K., Turner L.R., Mermelstein R.J., Flay B.R. (2005). Adolescents’ smoking expectancies: Psychometric properties and prediction of behavior change. Nicotine Tob. Res..

[32] Unger J.B., Soto D.W., Leventhal A. (2016). E-cigarette use and subsequent cigarette and marijuana use among Hispanic young adults. Drug Alcohol. Depend..

[33] Morello P., Perez A., Pena L., Lozano P., Thrasher J.F., Sargent J., Mejia R. (2016). Prevalence and predictors of E-cigarette trial among adolescents in Argentina. Tob. Prev. Cessat..

[34] Jackson C., Henriksen L., Foshee V.A. (1998). The Authoritative Parenting Index: Predicting health risk behaviors among children and adolescents. Health Educ. Behav..

[35] Perikleous E.P., Steiropoulos P., Paraskakis E., Constantinidis T.C., Nena E. (2018). E-Cigarette Use Among Adolescents: An Overview of the Literature and Future Perspectives. Front. Public Health.

[36] McCabe S.E., West B.T., Veliz P., Boyd C.J. (2017). E-cigarette Use, Cigarette Smoking, Dual Use, and Problem Behaviors Among, U.S. Adolescents: Results from a National Survey. J. Adolesc. Health.

[37] Dir A.L., Bell R.L., Adams Z.W., Hulvershorn L.A. (2017). Gender Differences in Risk Factors for Adolescent Binge Drinking and Implications for Intervention and Prevention. Front. Psychiatry.

[38] Lal R., Deb K.S., Kedia S. (2015). Substance use in women: Current status and future directions. Indian J. Psychiatry.

[39] Scheier L.M., Botvin G.J., Diaz T., Griffin K.W. (1999). Social skills, competence, and drug refusal efficacy as predictors of adolescent alcohol use. J. Drug Educ..

[40] Park S., Lee H., Min S. (2017). Factors associated with electronic cigarette use among current cigarette-smoking adolescents in the Republic of Korea. Addict. Behav..

[41] Perelman J., Alves J., Pfoertner T.-K., Moor I., Federico B., Kuipers M.A.G., Richter M., Rimpela A., Kunst A.E., Lorant V. (2017). The association between personal income and smoking among adolescents: A study in six European cities. Addiction.

[42] Piñeiro B., Correa J.B., Simmons V.N., Harrell P.T., Menzie N.S., Unrod M., Meltzer L.R., Brandona T.H. (2016). Gender differences in use and expectancies of e-cigarettes: Online survey results. Addict. Behav..

